# Applicability of factory calibrated optical particle counters for high-density air quality monitoring networks in Ghana

**DOI:** 10.1016/j.heliyon.2020.e04206

**Published:** 2020-06-16

**Authors:** C. Gameli Hodoli, F. Coulon, M.I. Mead

**Affiliations:** Cranfield University, School of Water, Energy and Environment, Cranfield, MK43 0AL, UK

**Keywords:** Atmospheric science, Environmental science, Geography, Earth sciences, Ghana, Sub-saharan Africa, Low-cost sensors, Air quality monitoring, Air pollution

## Abstract

In this study, we demonstrate the feasibility of using miniaturised optical particle counters (OPCs) for understanding AQ in Sub-Saharan Africa. Specifically, the potential use of OPCs for high-density ground-based air pollution networks and the use of derived data for quantification of atmospheric emissions were investigated. Correlation and trend analysis for particulate matters (PM), including PM_10_, PM_2.5_ and PM_1_ were undertaken on hourly basis alongside modelled meteorological parameters. Hourly averaged PM values were 500 μg/m^3^, 90 μg/m^3^ and 60 μg/m^3^ for PM_10_, PM_2.5_ and PM_1_, respectively and Pearson's correlation coefficient ranged between 0.97 and 0.98. These levels are in the agreement with range of PM emission reported for these types of environmental settings. PM was locally associated with low wind speeds (<= 2 ms^−1^) and was closely linked to anthropogenic activities. This study provides a benchmark for future AQ and demonstrates the feasibility of the current generation of OPCs for AQ monitoring in environments typical of large parts of West and Sub Saharan Africa.

## 1 Introduction

Air pollution is a major environmental risk globally to health with more than 90% of the world's population living in regions where AQ levels do not meet the 2017 World Health Organization (WHO) recommended thresholds (Health Effects Institute, 2019). Different components of air pollutants and their contribution to premature deaths have been documented by WHO (World Health Organization, 2006). The species with the strongest health-damaging effects were found to be, in order, particulate matter (PM), ozone (O_3_) and nitrogen dioxide (NO_2_) (World Health Organization, 2006). PM is identified globally as a risk indicating factor and often used as a key indicator of urban AQ (Cohen et al., 2005). Across Africa, 520 million children are reported to be exposed to polluted air (UNICEF, 2016). Additionally, global climate change is projected to have a significant adverse effect on Africa and is intrinsically linked to air pollution (Ramanathan and Feng, 2009). This implies that AQ policies would be most effective if they are linked to climate change policies. Understanding atmospheric emission sources in urban settings across Sub-Saharan Africa is important for understanding and moderating short to medium term AQ dis-benefits as well as for modelling climate change (Melamed et al., 2016). Of particular interest in this study are the relatively high levels of PM emissions resulting from combustion (including vehicular) as identified by the Ghana Environmental Protection Agency (GhEPA) as the main pollutant of interest in Ghana (GhEPA personal communication, 2018). In Ghana similar to other parts of SSA, major sources of air pollution are traffic-related associated with increased vehicle fleets, use of solid fuel, improper waste management practices and the slash and burn agricultural practices (Health Effects Institute, 2019; Schwela, 2012; Dionisio et al., 2010).

**Figure 1:**
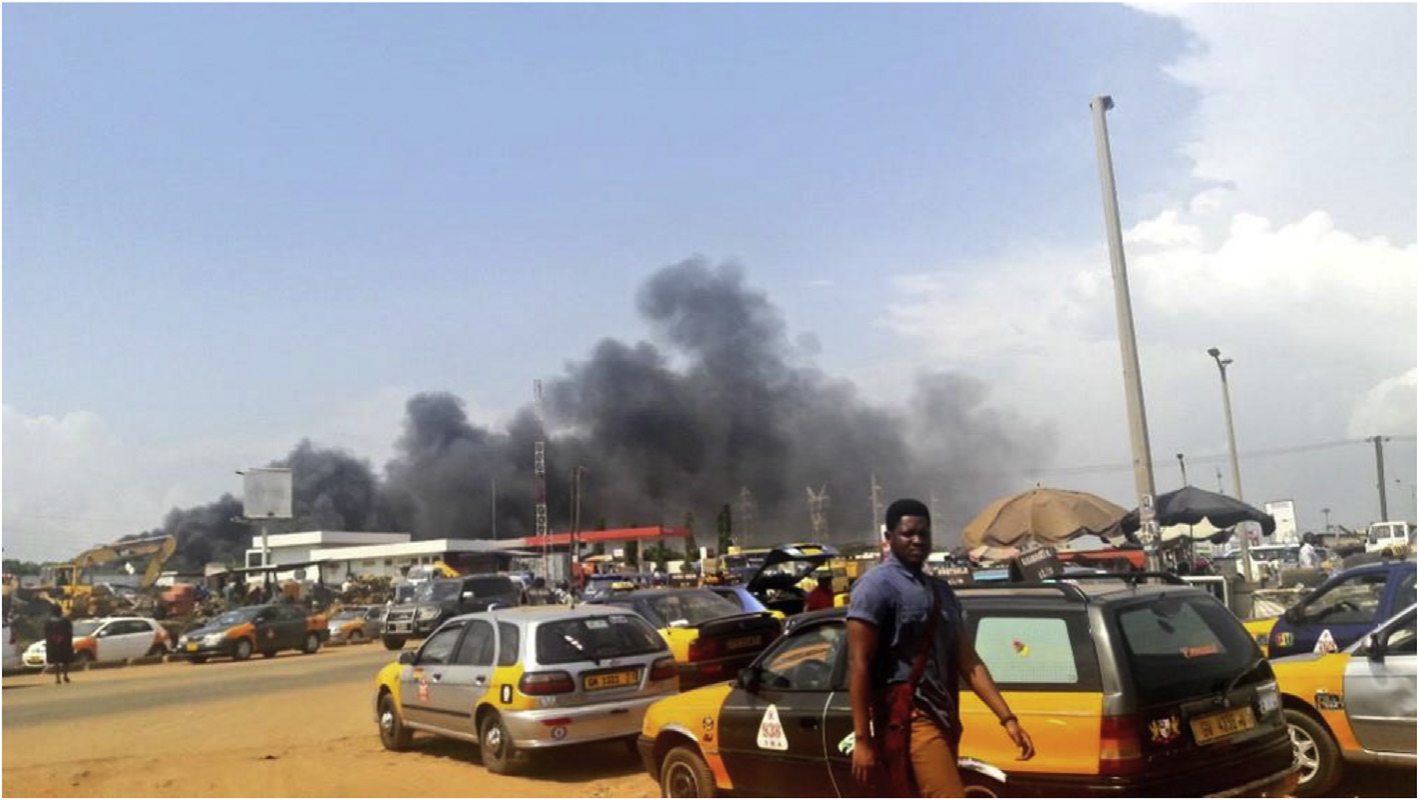
Typical Ghanaian setting showing the complex and varying emission sources.

Many sub-Saharan African (SSA) countries, including Ghana, have limited capacity for undertaking AQ monitoring and a significant number lack AQ standards (Petkova et al., 2013). Where available monitoring is undertaken by countries such as Ghana rely on conventional monitoring approaches which require significant infrastructure, routine maintenance and periodic calibration (Schwela, 2012). AQ monitoring (AQM) is therefore undertaken sporadically or at a very limited number of sites (Schwela, 2012). Additionally, a large number of AQ monitoring projects initiated in SSA are discontinued after approximately 1 year due to maintenance and operational issues (Schwela, 2012). For example, GhEPA has previously partnered with a number of international organisations and academic institutions since 2005 to undertake AQ studies. However, most of these studies did not include any training of staff and the sustainability of these programmes was often overlooked with limited long-term planning for operation beyond the life of specific projects (GhEPA personal communication, 2018). For example at Cape Coast (study area), no regulatory air quality monitoring network or preliminary air quality campaign has been established on the applicability of new technologies for AQ monitoring similar to other parts of Ghana (i.e. as at the period of deploying the two AS510 static multi-sensor nodes; see Table 1 in sub-section 2.1) except Accra the capital city where some LCS have been deployed (GhEPA personal communication, 2018). Main background activities in these areas include traffic, roadside food vending including use of solid fuel for cooking, unpaved road networks, commercial markets (Figure 1).

**Table 1:** Summary of technical characteristics of the AS510 static multi-sensor node with details of the OPC-N2.

Measurands/Activity		Technology
Particle size distribution		Optical particle counter (OPC)
CO, NO, NO_2_ and O_3_		Electrochemical (ECs)
VOCs		Photo ionization (PID)
CO_2_		Non-dispersive infra-red (NDIR)
T and RH		Capacitive
Timestamp and location		Global positioning system (GPS)
Data telemetry		General packet radio service (GPRS)
OPC-N2 details	Particle range	0.38–17 μm
	Data bins	16
	Flow rate	1.2 L/min
	Sample flow rate	220 mL/min

Depending on instrument siting and network operational cycle actual local ambient levels of pollutants can vary significantly spatially and in time when compared to hourly measured levels at nearby sites (e.g. Mead et al., 2013; Moltchanov et al., 2015; Kumar et al., 2015; Broday et al., 2017). GhEPA undertakes routine AQ monitoring at a limited number of sites. Its network is made of 16 sites in the capital city of Accra (the largest urban area in Ghana). All sites measure ambient particulates using gravimetric samplers. The instruments used (one at each site) are a high volume cascade impactor (Andersen Impactor, Tisch Environmental Inc., USA) and a mini volume sampler (MiniVol Portable Air Sampler. Airmetrics, USA). These are used to manually sample AQ data for PM_10_ and PM_2.5_ species respectively. Each site produces a single 24 h averaged sample once every 6 days. This limited approach is not statistically sufficient to analyse and assess daily variation of PM and does not provide information on other important species such as O_3_, carbon monoxide (CO), the oxides of nitrogen (NO + NO_2_ = NOx) or volatile organic compounds (VOCs) (e.g. Moltchanov et al., 2015; Kumar et al., 2015; Broday et al., 2017). Greenhouse gases such as carbon dioxide (CO_2_) are also not monitored. This lack of speciated or appropriate resolution data in Ghana and wider SSA limits accurate assessment of human health exposure to air pollution in the region. Limited local AQ data limits understanding of local pollution levels. As a result, development, implementation and evaluation of location-specific, targeted air pollution control measures are limited if not unattainable (Petkova et al., 2013).

The low-cost sensor offers the opportunity to solve some of these issues by providing a valuable AQ monitoring capability in resource-constrained settings (Mead et al., 2013; Snyder et al., 2013; Castell et al., 2017). Most current-generation commercial low-cost sensor nodes from recognised manufacturers are user-friendly have low power requirements and will routinely telemeter data from site to remote data repositories. Most are capable of collecting high spatiotemporal resolution data making them suitable for detailed quantification of the spatial and temporal variability of ambient air pollutants (Mead et al., 2013; Snyder et al., 2013; Castell et al., 2017). In SSA and other developing regions, minimal additional infrastructure including security and technical training are needed to deploy low-cost sensor nodes for AQ studies. However it should be noted that there is still the requirement for skilled development of optimised deployment strategies and back end analysis of collected data, particularly for the scale of data this class of sensors are capable of generating (Snyder et al., 2013; Seto et al., 2014; Kumar et al., 2015). Critically this class of sensor nodes have been shown to require site-specific calibration techniques and approaches. In the majority of case, their utility is linked to the availability of local reference data.

In this proof of concept study, the applicability of emerging low-cost air sensors in Ghana as a widely applicable case study for the wider SSA was investigated based on an 8-week field deployment. Specifically, the extent to which the deployed devices can be used to provide independent high-level resolution speciated data for PM and inform decision making on AQ in such resource-constrained settings was investigated. The study relied on the inbuilt data correction algorithms developed by the sensor manufacturer to test the potential these types of sensors offer expanding ground-based spatial AQM in these types of environments. No further data correction mechanisms were applied though temperature and relative humidity have shown to introduce inconsistencies in the LCS reported data (see e.g. Hagler et al., 2018; Malings et al., 2019; Snyder et al., 2013; Moltchanov et al., 2015). This work seeks to bridge the huge scientific knowledge gap on the feasibility of LCS specifically under a wide variety of climate regimes such as those encountered in SSA, ranging from humid climate in the tropics to arid and semi-arid climate in the sub-tropics.

## 2 Methods

In this study, we focused on testing the utility of LCS to understand the extent to which factory calibrated Alphasense OPC-N2 to provide reliable ground-based air quality data in environments previously unachievable due to cost and logistical requirements. As a proof of concept approach, no data correction mechanisms were applied to improve or evaluate the accuracy of the selected LCS. The study, however, showed that LCS in their current form are capable of quantifying atmospheric emissions and the high-resolution data could provide meaningful insights for air pollution management tasks such as developing, implementing and tracking air pollution mitigation strategies though further research is required to confirm this.

### 2.1 Instrumentation

Two AS510 multi-sensor nodes (Atmospheric Sensors, UK), were used for this study. These nodes measure: CO, NO_x_, O_3_, VOCs, PM and key environmental parameters relative humidity (RH) and temperature (T). Table 1 lists the species measured and the technologies used for these measurements.

The resolution of the nodes used in this study for all measured species was 60 s for the duration of the study. This study is focused on particulates and details of the Optical Particle Counter (OPC) component of the node is also presented in Table 1. The OPC (Alphasense, UK OPC-N2) measures scattered light from particulates from the sampling beam to reconstruct particle mass levels (Hinds, 1999). For a detailed description of the OPC design and operation see Alphasense reference note (OPC-N2 Monitor, Alphasense Ltd UK, 2015).

### 2.2 Site selection and data acquisition

**Figure 2:**
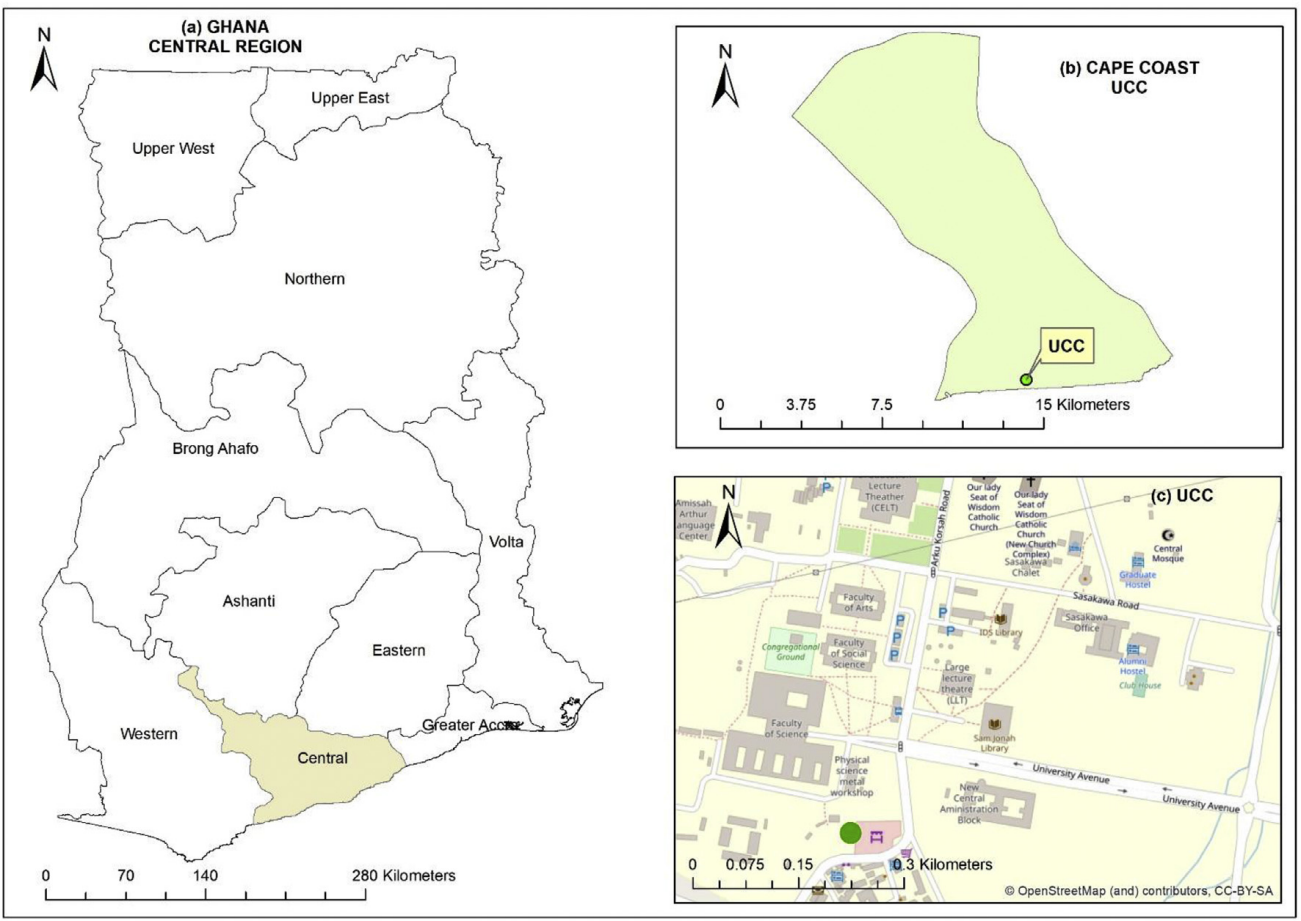
Overview of the deployment area at the University of Cape Coast (UCC) (a. overview of the central region location; b area covered by UCC; c. street view nearby the UCC). The green circle shows the location of the two co-deployed nodes ~10 cm apart (05°06′N 01°15′W).

Both AS510 multi-sensor nodes were co-deployed at a central site at Cape Coast, Ghana. This site was selected as being typical of expanding urban settings outside of Accra with a broadly similar composition as other urban areas of this type. Co-deployment was for 6 weeks (August 9^th^ to September 18^th^, 2018; Figure 2) and provided a baseline for comparison of data between the sensor nodes. Cape Coast is situated in the South of the country on the Gulf of Guinea with a population of approximately 170000 (GSS, 2012). The region is relatively humid with mean monthly relative humidity (RH) ranging between 85% and 99% as compared with a range of 77%–85% in Accra. The predominant wind direction at Cape Coast is from the south which has the potential to transport pollutants from across the region to Cape Coast as well as for onshore relatively clean air masses to be transported. Nodes were mounted 10 cm apart at a height of 4 m above the ground. Typical sources in the area include unpaved roads (re-suspended dust), road-side food preparation (biomass and gas combustion), taxi ranks (vehicular) and roads used by private vehicles as well as heavy trucks and commercial vehicles.

**Figure 3:**
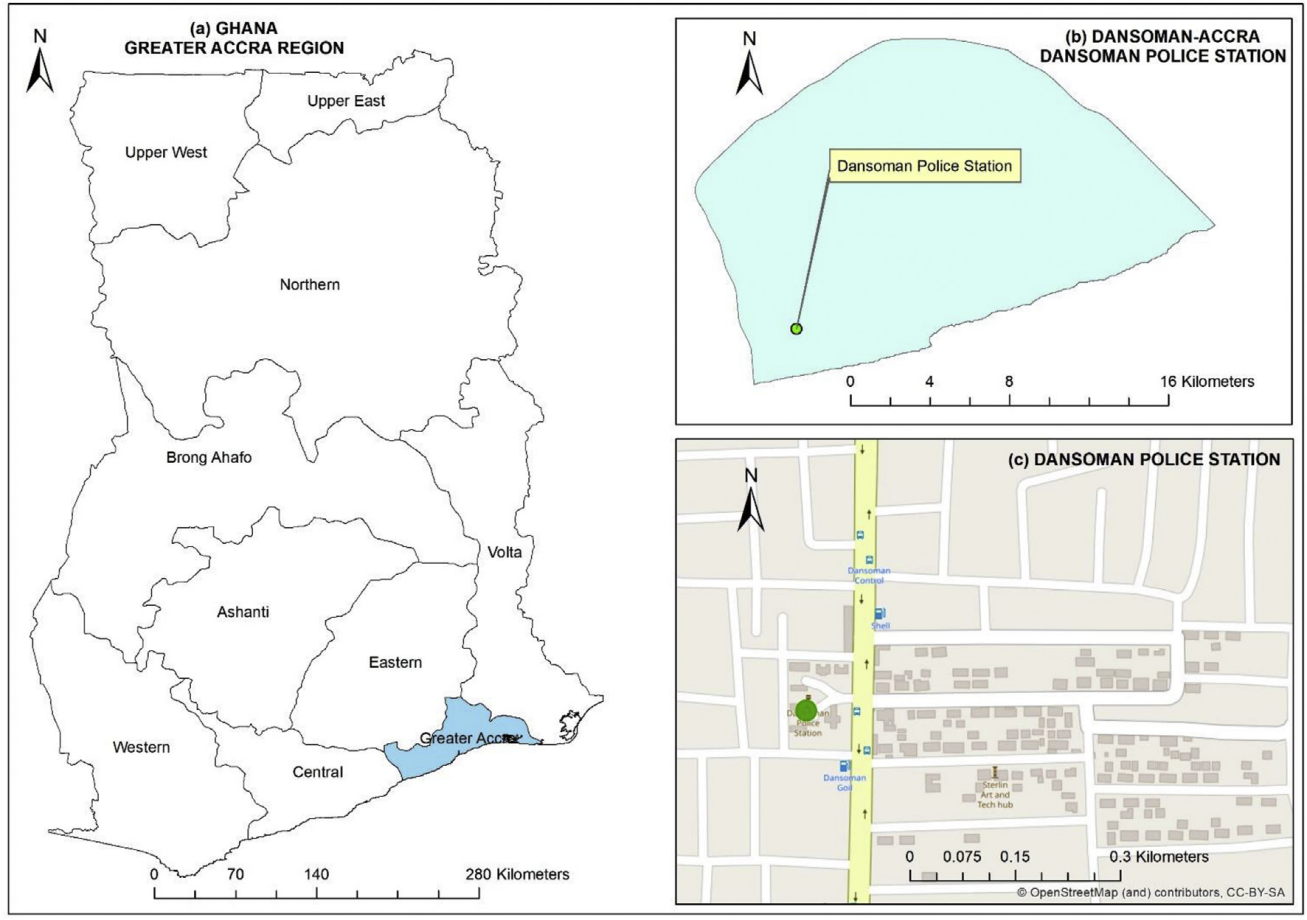
Overview of the Dansoman-Accra site deployment (a. overview of the greater Accra region; b area of the Dansoman police station; c street map overview nearby the Dansoman police station; Green circle: location of the node collocated ~10 cm apart with the GhEPA monitoring reference device) GhEPA (5°32′28″N 0°16′8″W).

After completion of the initial 6 weeks co-deployment measurement period, one node was relocated to central Accra (approximately 147 km north of Cape Coast) alongside the GhEPA reference high volume sampler used for monitoring PM10 is located (Figure 3). The purpose of this collocation is to understand the precision of the selected OPC-N2 for PM monitoring and potentially develop data correction mechanisms for their use in subsequent publications. This data is therefore not presented but reported LCS data from the two nodes at Cape Coast as an experimental study. Accra covers approximately 225.67 km2 with a population of 2.5 million (GSS, 2012) and is the economic and industrial capital of Ghana. The node was moved there as a study investigating the potential for cross-validation of sensors or radically different operational cycles. This type of low resolution, low technical overhead PM monitoring is more widespread than online routine PM monitoring across the region (Health Effects Institute, 2019). The site is a residential area close to the relatively high use Dansoman Highway, a local open market (including open food preparation), a fuel station and more dispersed road-side food vendors.

Due to the limited availability of local meteorological data, modelled wind data from the Global Forecast System (GFS) repository was used for source apportionment in this study (NOAA, 2019; Fernández-López and Schliep, 2019). The GFS is a dataset from the National Oceanic and Atmospheric Administration (NOAA) and the National Centers for Environmental Prediction (NCEP) (Fernández-López and Schliep, 2019). Within this database, wind data since 2011 is saved at 3-hour intervals daily in velocity vector format with a resolution of 0.5° and ~50 km.

**Figure 4:**
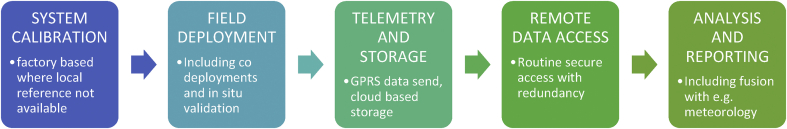
Schematic overview of novel reproducible protocols developed and employed for use of factory calibrated miniaturised OPCs in environments with limited AQ monitoring capabilities.

A step by step approach adopted in this study for data acquisition and analysis is presented in the chart in Figure 4. All analysis was performed using the “openair” package including sectorial plots in the function of wind components, trend and cluster analysis as well as Pearson's correlation analysis. In-depth information on these packages used are not provided in this study but further details can be found here Carslaw (2019).

## 3 Results and discussion

Though there are still questions regarding the accuracy of low-cost sensors (Lewis et al., 2016), their ability to obtain high-resolution spatiotemporal data makes them suitable for extending air quality monitoring networks. For Crilley et al. (2018) reported that the consistency between the current generation of OPCs make them applicable for understanding the spatial variability of PM species. On data quality, relative humidity beyond 85% has been found not to significantly affect the reported OPC data (Crilley et al., 2018). Spinelle et al. (2017) also found no impact of temperature and relative humidity on reported OPC-N2 data. The reported data used in this study met these preliminary requirements making it useful for air pollution management strategies. For example, Snyder et al. (2013); Rai et al. (2017), Jerrett et al. (2005) and Castell et al. (2017) have shown that the reported data can be used to establish the link between atmospheric exposure to human health, emergency response management, community's awareness of air pollution and complement regulatory air quality monitoring stations. The results presented in this publication is based on a proof of concept study and to bridge the huge scientific knowledge gap on the utility of LCS for AQM in Ghana and wider SSA. As briefly stipulated in the introductory part of this study, ground-based AQM is rudimentary in SSA but the emergence of LCS offers a unique and alternative approach. Air pollution studies using LCS in advanced countries e.g. Europe and Americas have reported some caveats, for example, poor data quality due to the impacts of environmental variables such as temperature and relative humidity (Barron ad Saffel, 2017; Malings et al., 2019; Snyder et al., 2013; Kumar et al., 2015; Hagler et al., 2018) and the impacts of hygroscopic growth on PM species quantification (Malings et al., 2019). A number of studies have also shown that machine learning approaches and sophisticated mathematical models for example regression can be used to improve LCS data quality. In some cases, commercial data mining and improvement tools have been developed for end-users (e.g., Advanced Normalization Tool for AirVision, 2020; Hagler et al., 2018). None of these applications has been used in this preliminary study but to rely on inbuilt data correction mechanisms thereby selecting temperature corrected values from the reported LCS data deployed in Ghana. Key considerations that influenced the approach documented here is whether LCS in their current form will function under the harsh environmental conditions coupled with complicated emission sources in Ghana to obtain reliable AQ data when deployed under field conditions in these types of environment as well as how the reported data can be used to spur regulatory action. The results of this field campaign though not corrected have shown that low-cost PM sensors are capable of providing background information on PM levels specifically in highly polluted environments as echoed by Castell et al. (2017) and are capable of providing a high-density network to understand spatial and temporal variability of PM species. For example in an experimental study by de Souza et al. (2017) and the use of Alphasene OPC-N2 for monitoring traffic pollution by Pope et al. (2018) similar findings were observed. It is deduced from this preliminary finding in Ghana that the homogeneity of these environments (Ghana and wider SSA) presents a unique opportunity for further studies to develop a generalized data correction mechanism for the use of the selected sensors in these environments.

### 3.1 Sensor intercomparison (PM)

**Figure 5:**
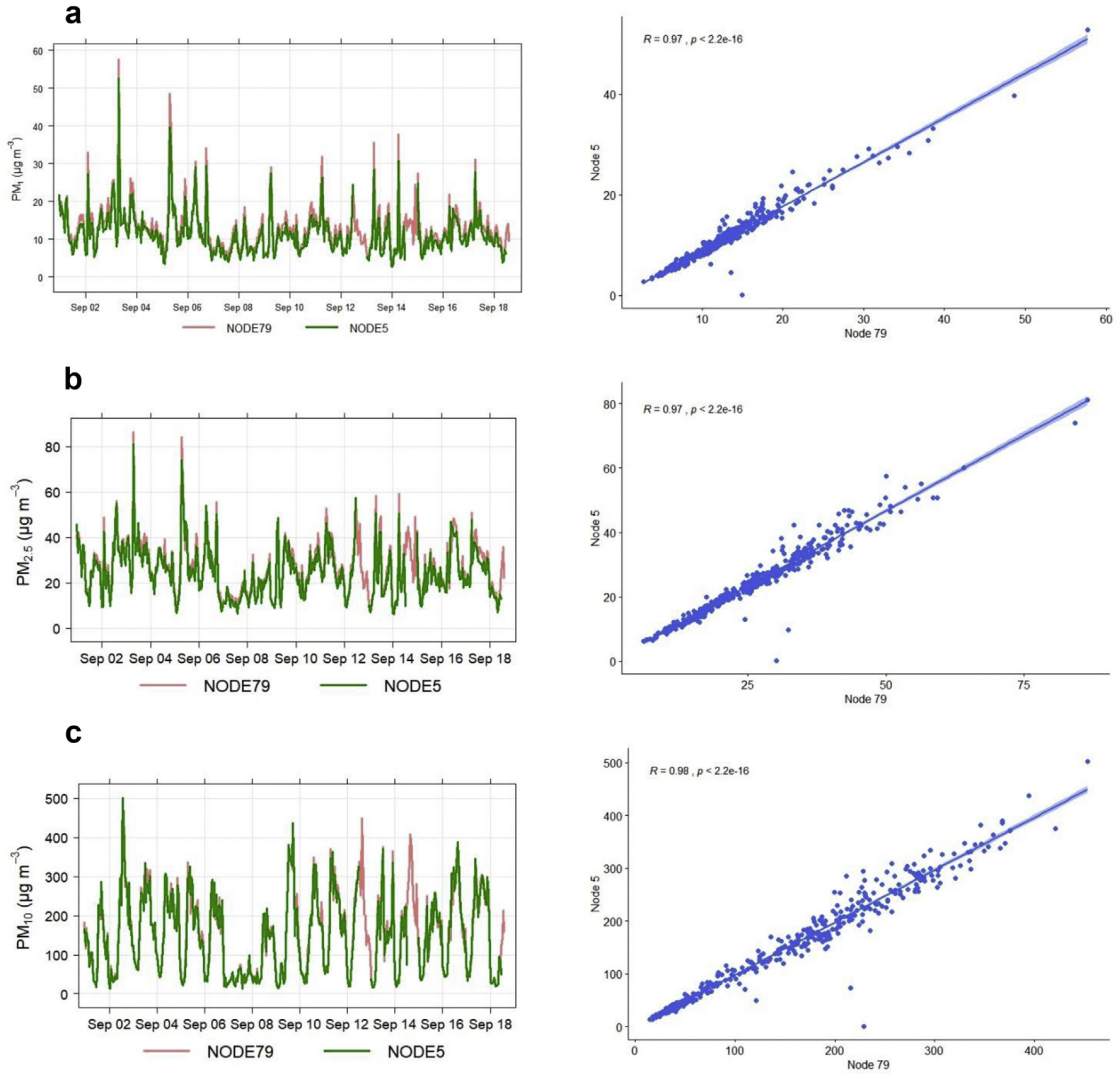
Hourly time series and corresponding Pearson correlation plot of data from Node 79 versus Node 5 at UCC: (a) PM_1_, (b) PM_2.5_, and (c) PM_10_.

Hourly averaged PM (PM_10_, PM_2.5_ and PM_1_) data from the selected two nodes during the deployment at Cape Coast (i.e. UCC site) showed that the reported data from the nodes are highly reproducible as the signal acquisition of the two nodes is similar (Figure 5a, b and c) with corresponding Pearson's correlation analysis (R) of 0.97, 0.97 and 0.98 for PM_1_, PM_2.5_ and PM_10_, respectively. The first 3 weeks of deployment have not been included in this analysis as issues with data telemetry led to limited data for the study.

The mean PM values of the two nodes are significantly different. Comparing the mean values of each of the PM categories from the two devices and their corresponding *t-values* it can be seen that this statistical difference (see Table 2) reduces for PM categories in the order of PM_1_, PM_2.5_ and PM_10_. Additionally, since PM_10_ values > PM_2.5_ values > PM_1_ values as demonstrated in the statistical difference between the PM species indicates that low-cost PM sensors are suitable for coarse particle monitoring in these types of environments as compared to fine particles but further studies are required to support this preliminary claim. This finding, however, is in agreement with previous reports using these types of sensors, for example, Castell et al. (2017).

**Table 2:** Mean and standard deviation of PM in μg/m^3^ with t and p-values showing the statistical difference between the two nodes at UCC.

Species	Node 5		Node 79		Statistical difference	
	Mean	SD	Mean	SD	*t*	*p-value*
PM_1_	11.4	8.9	12.9	10.1	17.3	<2.2e-16
PM_2.5_	24.7	19.7	26.8	21.3	11.4	<2.2e-16
PM_10_	149	175.1	156.6	179.2	4.2	1.9e-5

### 3.2 Wider comparisons (PM)

These findings are in agreement with the assertion that current OPCs require optimisation (e.g. application of machine learning/post data correction with sophisticated mathematical models) for measuring fine particles since they measure particles lager than 0.3 μm. Also, the statistical difference between the two nodes from the same manufacturer with p-value <0.05 echoed the challenges on the use of low-cost sensors, for example, depending on inbuilt correction algorithms which is mainly influenced by time and resources invested by the manufacturer (Baron and Saffell, 2017).

PM_10_ concentrations peak at 500 μg/m^3^. This is in agreement with levels recorded in other polluted environments (Wang et al., 2015) and SSA (Brauer et al., 2012; Health Effects Institute, 2019). Though these pilot findings are in agreement with levels of PM pollution recorded in such environments specifically Ghana, limited studies are using these types of low-cost sensors for comparison and justification. Studies with emerging low-cost sensors have shown that low-cost sensor technologies suffer environmental artifacts namely relative humidity and temperature thereby affecting the measured data and do not agree well with measurements from instruments using different measurement technologies/principles (Watson et al., 1998; Chow et al., 2008). For example, Zheng et al. (2018) found that low-cost PM_2.5_ sensor Plantower model PMS3003 corresponds very well with a scattered light spectrometer (r of 0.8) versus low correlation with a beta attenuation monitoring (r of 0.5). These findings, however, provide a benchmark for future studies with these types of low-cost sensors especially in developing data correction/validation and calibration procedures for the use of low-cost sensors for AQ monitoring in Ghana and similar environments.

### 3.3 Using LCS data for tracking location-specific air quality standards

**Figure 6:**
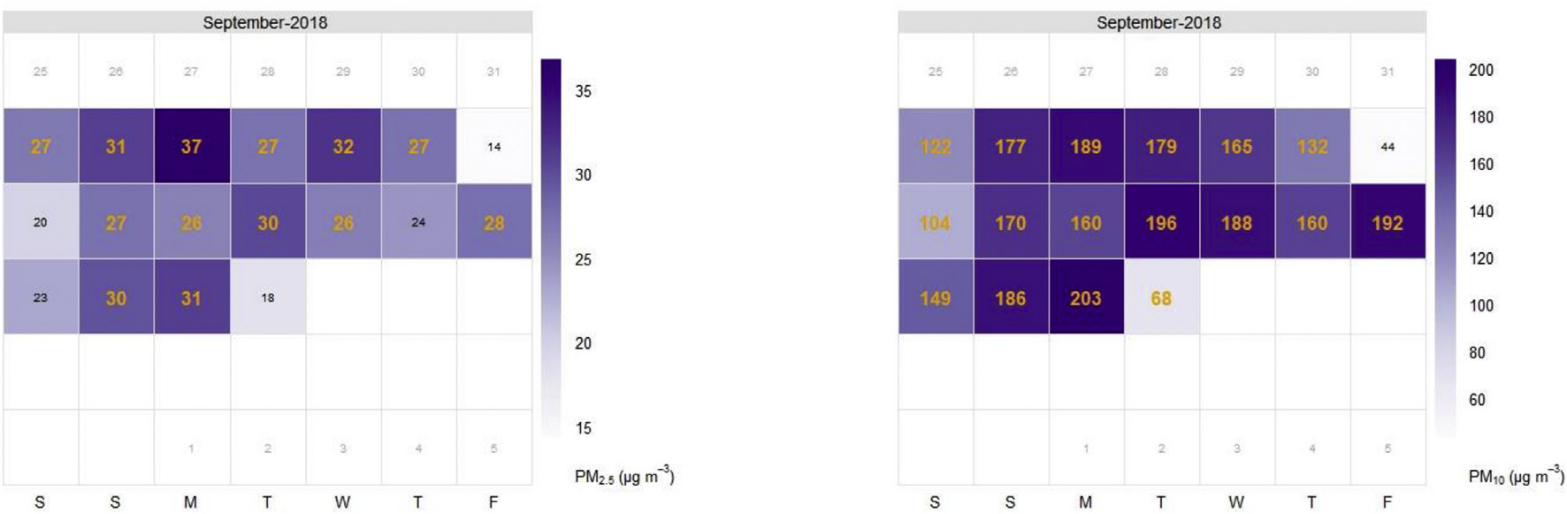
Calendar plot of PM at UCC for September 2018 showing potentials of comparing reported data to location-specific regulatory standards e.g. WHO daily mean values (25 μg/m³ for PM_2.5_ and 50 μg/m³ for PM_10_) if validated. Dark orange values represent days where the daily guidelines were exceeded.

The reported data plotted in a calendar format demonstrate the potentials LCS offer in reporting high-resolution routine and site-specific data suitable for tracking air pollution regulations (Figure 6). Comparisons can be drawn using data from these types of sensors if the reported data is improved/validated. Rai et al. (2017) has reported that LCS does offer the opportunity to increase a community's awareness of air pollution and help track exposure to human health as well as support emergency responses. The capability of LCS to obtain routine site-specific data which can be quantified with data mining approaches as demonstrated in this calendar plot is useful for air pollution control specifically in environments with limited knowledge on air pollution and its adverse health impacts such as Ghana and wider SSA.

Here, we experiment this approach by comparing the reported data to current WHO AQ guideline values of 25 and 50 μg/m³ for PM_2.5_ and PM_10_, respectively for September 2018 (Figure 6). These thresholds were exceeded. Even though the reported data from the AS510 nodes used in this study is not validated with data from site-specific reference equipment, the PM levels reported are in agreement with levels expected and recorded previously in SSA (Amegah, 2018; Health Effects Institute, 2019) and have shown that high-temporal data from low-cost PM sensors are suitable for tracking air quality guidelines and inform decisions. Further to this, this type of analysis is currently unachievable with the GhEPA monitoring regime as only 24-hour averaged data can be collected roughly 5 times a month.

### 3.4 PM trends

**Figure 7:**
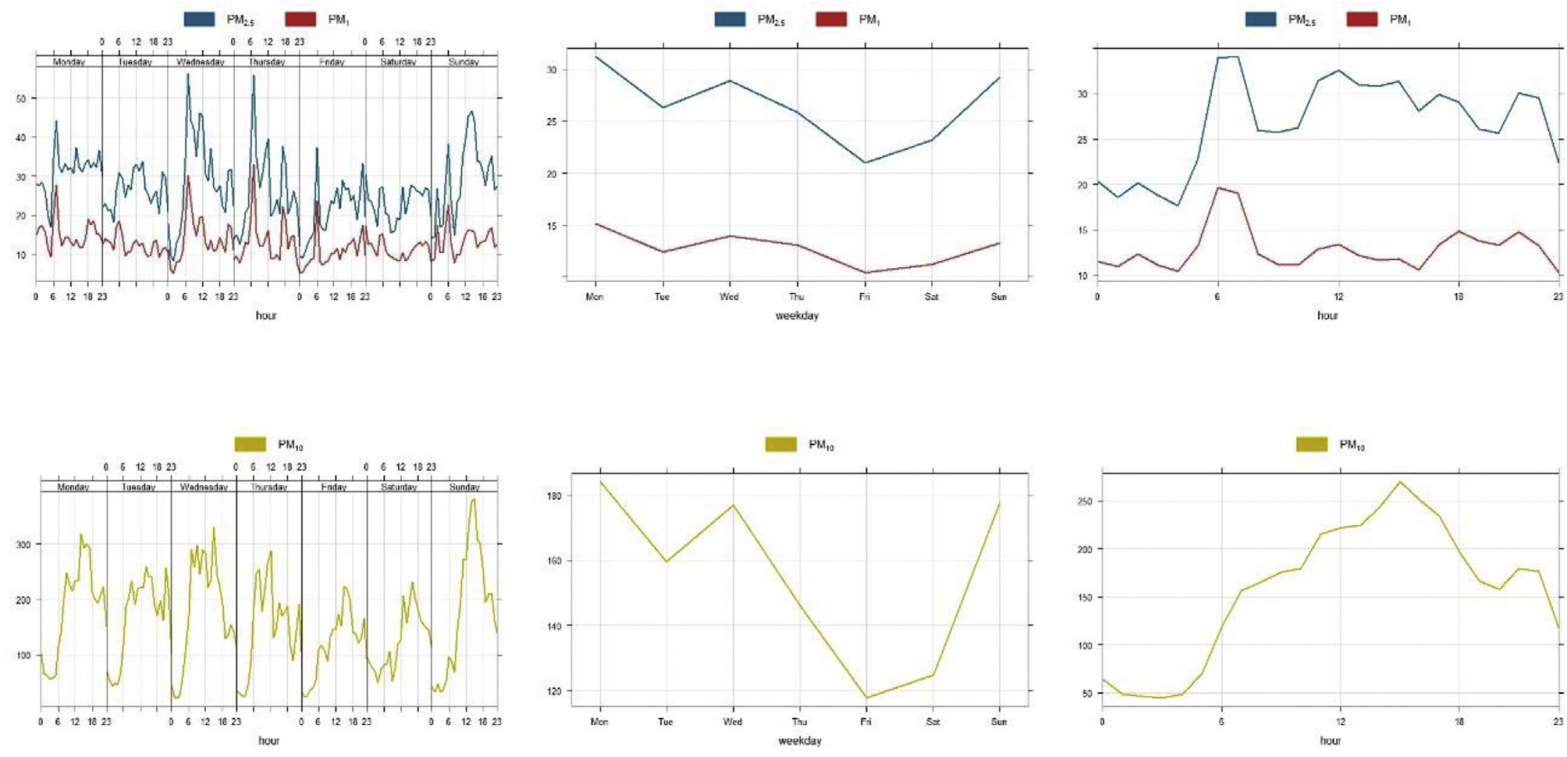
Trends of PM_1_ and PM_2.5_ (top) and PM_10_ (bottom) by hour and day of the week (left), by weekday (centre) and by hour of the day (right) at the UCC sampling site.

Trends of PM species showed peak levels in the mornings which are attributable to typical sources such as unpaved roads (resuspended dust), road-side food vendors (biomass and hydrocarbon combustion), taxi ranks (tailpipe) and roads used by heavy trucks and commercial vehicles (Figure 7). Urbanisation coupled with increasing motorization is indeed a major source of air pollution in SSA (Petkova et al. (2013) Schwela, 2012a; Amegah and Agyei-Mensah, 2017). A drop in PM level was observed on Friday which is attributable to reduced human activities and peaks again on Sundays (Figure 7 left bottom and top panel) due to increased anthropogenic activities. Though this is not documented, Ghanaians are identified as highly religious people hence the high specks of PM levels on Sundays is attributable to motorization for religious activities specifically church activities. This does require further studies as meteorological parameters are influential in atmospheric emissions. These findings are unachievable with conventional and sparsely distributed AQ monitoring stations (e.g. in Ghana, data is averaged 24 h and collected every 6 days). Also, understanding the complexity of emission sources in urban areas requires monitoring at fine scales (Jerrett et al., 2005; Karner et al., 2010; Eeftens et al., 2012) and ability to potentially establish a dense network without huge infrastructure. Low-cost sensors offer these opportunities and can be used in resource-constrained settings (Snyder et al., 2013; Mead et al., 2013; Castell et al., 2017).

### 3.5 Local pollutant sources

**Figure 8:**
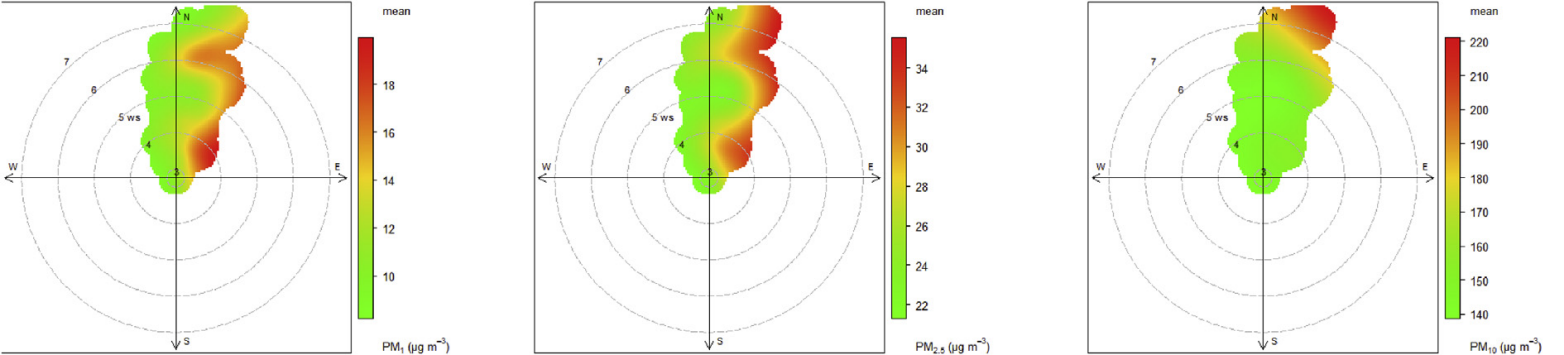
Primary source apportionment of PM; left panel PM_1_, middle panel PM_2.5_ and right panel PM_10_ using hourly bivariate polar plot at the UCC site.

Polar plots were used to identify the sources of monitored species based on the high-resolution data from the low-cost devices (Figure 8) for this period. The trend between PM_1_ and PM_10_ suggests that an important source of particulate matter is located towards the NNE. This source is either biased towards lighter particles or that larger particles are removed before arriving at the monitoring site. The data also potentially points towards a more local source of lighter particulates nearer to the monitoring site which has an important role in composition at lower wind speeds. Under still conditions, it seems there is no significant local source. Overall PM levels were relatively high (20 μg/m^3^ for PM_1_, 35 μg/m^3^ for PM_2.5_ and 220 μg/m^3^ for PM10 as compared to the recommended 25 μg/m^3^ and 50 μg/m^3^ limits of the WHO for PM_2.5_ and PM_10_ respectively). Locally PM_1_ and PM_2.5_ concentrations were high while high PM_10_ concentrations were experienced at higher wind speed.

These results reflect that both nodes were installed (at the UCC site) a few meters away from the main road and in a traffic dominated area. The region to the NNE is a mostly unpaved flat area close to the Gulf of Guinea. The nature of the deployment site (unpaved roads with associated resuspended wind-blown dust) coupled with the topography of the area (a relatively flat field) would be expected to have contributed to higher PM_10_ levels with increased wind speed. Especially considering that the area to the NNE is dominated by unpaved road, win-blown dust and sea salt from the nearby coast. As it has been shown that coarse PM dispersion is linked to higher wind speed (Carslaw and Ropkins, 2012) we would expect a reduction in the PM_10_ signal at lower wind speeds (2–5 m/s) and higher levels were observed at higher wind speed (6–8 m/s).

### 3.6 PM trend between two socio-economic settings

**Figure 9:**
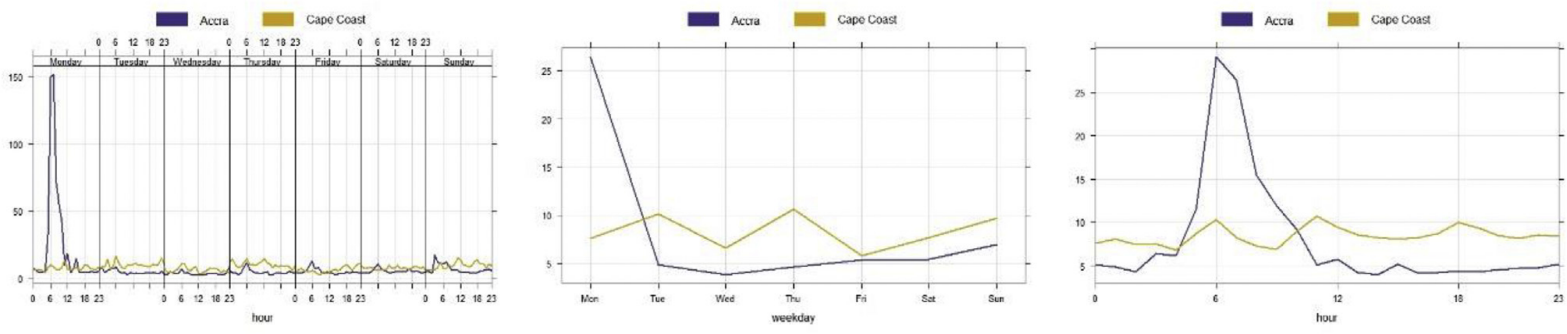
Trend plots for PM_1_ at Dansoman-Accra and UCC-Cape Coast: left panel – day of week, middle panel – by weekday and right panel – hour of day.

In Accra, peak values of PM_1_ were observed on Monday which then drastically reduced to a concentration below 50 μg m-3 (Figure 9). This preliminary finding could be linked to emissions from background activities such as garbage burning, vehicular emissions or linked to the functionality of the deployed device. In a study to understand the patterns of air pollution in the neighbourhoods of Accra, it was observed that poorer households are highly exposed to air pollution. This in part is due to the use of biomass and/or solid fuel as a source of energy for heating and cooking (Dionisio et al., 2010).

Apart from Monday and Friday, PM_1_ concentrations remain relatively high at Cape Coast (Figure 9), a relatively poor socio-economic setting is potentially linked to this assertion; energy source (use of biomass and/or solid fuel as a source of energy for heating and cooking) as compared to Accra. Though higher PM level is expected because of the nature of the deployment site; near the Dansoman Highway and a residential area and mini refuse damp (e.g. garbage is sometimes burnt during cleaning activities including car tyres), further research is required to provide a better understanding of this finding since the peak occurs on a single day (Monday). Monday morning peaks (rush hour) were not observed at Cape Coast as compared to Accra, the concentrations remained moderately higher for the rest of the period except for Friday.

## 4 Conclusions and recommendations

This study has shed light into the feasibility of factory calibrated OPCs for quantifying and understanding sources of PM species and highlighted how low cost sensors can assist countries from West Africa and wider SSA in establishing a high density of ground-based AQ networks to understand spatial and temporal variability of PM species. It has demonstrated the applicability of the high-resolution data for air pollution management and community engagement by developing analytical tools for data management, visualization, analysis and interpretation. It further showed that low-cost devices are suitable for air quality monitoring in environments with limited or no regulatory air quality monitoring stations. They can be used to track and evaluate exposure levels, understand emission trends, define pollution level at varying locations with different background activities and suitable for providing reliable high-resolution data for source identification. Future studies need however additional focus on data correction/validation when using these types of sensors in these types of environments.

## Declarations

### Author contribution statement

C. G. Hodoli, Performed the experiments; Analysed and interpreted the data; Contributed reagents, materials, analysis tools or data; Wrote the paper.

Frederic Coulon: Conceived and designed the experiments; Analysed and interpreted the data; Wrote the paper.

M. I. Mead, Conceived and designed the experiments; Analysed and interpreted the data; Contributed reagents, materials, analysis tools or data; Wrote the paper.

### Funding statement

C. G. Hodoli was supported by the Ghana Education Trust Fund (GETFund).

### Competing interest statement

The authors declare no conflict of interest.

### Additional information

No additional information is available for this paper.
